# Supplementary material for: Compliance of Static Stretching and the Effect on Blood Pressure and Arteriosclerosis Index in Hypertensive Patients
Source: Clin Pract. 2022 May 9;12(3):306–17. doi: 10.3390/clinpract12030036 (PMC9149945; doi:10.3390/clinpract12030036)

Supplemental Figure S1. Stretching protocol

1. Calf

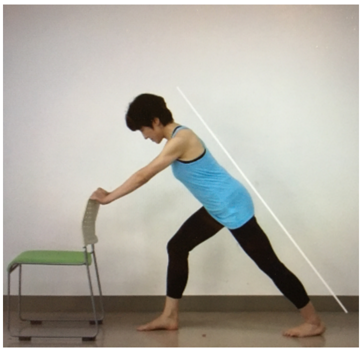

2. Back of the thigh

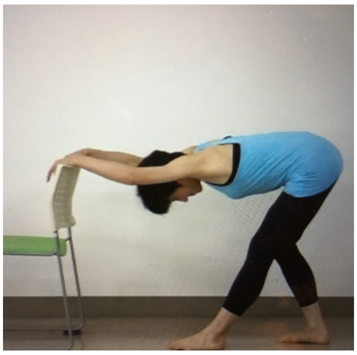

3. Front of the thigh

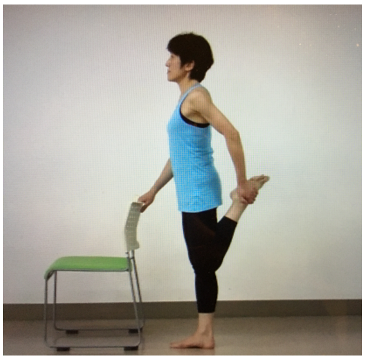

4. Inside of thigh

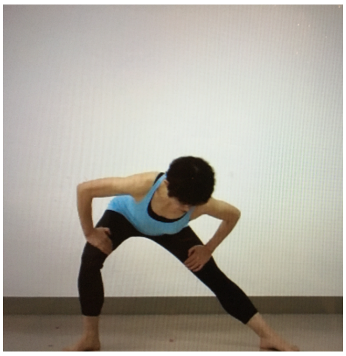

5. Waist

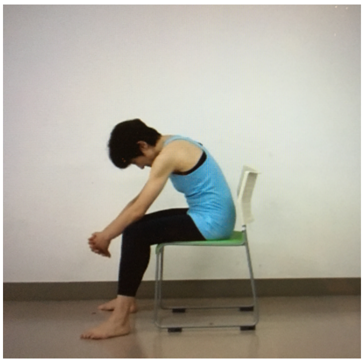

6. Upper back

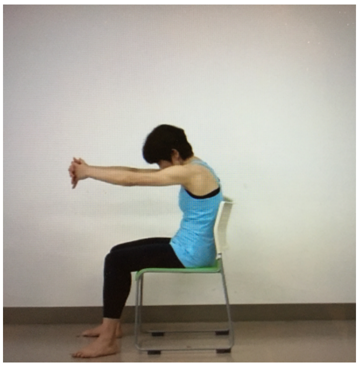

7. Neck

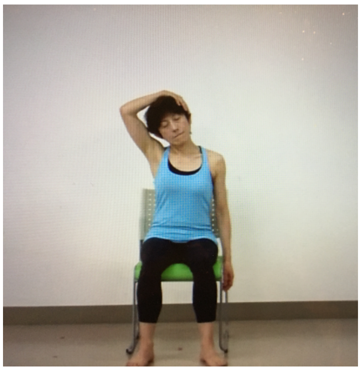

8. Shoulder

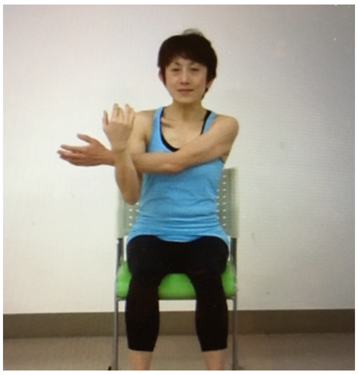

9. Upper arm

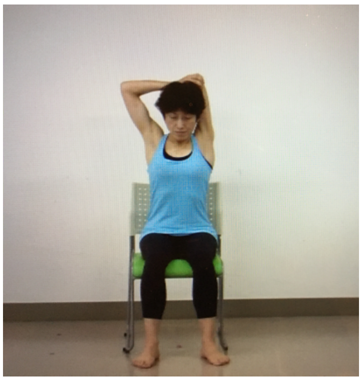

10. Wrist

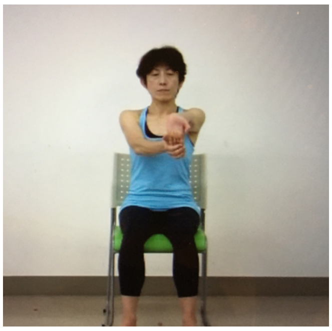

Supplement: Supplementary file 1 [file clinpract-12-00036-s001.zip › clinpract-1686423-supplementary.pdf]
